# Q Fever in Como, Northern Italy

**DOI:** 10.3201/eid1001.030467

**Published:** 2004-01

**Authors:** Domenico Santoro, Raffaele Giura, Maria Chiara Colombo, Paola Antonelli, Maria Gramegna, Oscar Gandola, Giulio Gridavilla

**Affiliations:** * St. Anna Hospital, Como, Italy; † Department of Prevention, Azienda Sanitaria Locale, Como, Italy

**Keywords:** Q fever, pneumonia rickettsia infecitons, disease outbreaks

**To the Editor:** Q fever is a widespread zoonosis caused by the intracellular gram-negative bacterium *Coxiella burnetii.* Infection in humans usually occurs through inhalation of contaminated aerosols from parturient fluids of infected animals or contaminated wool (*1*). *C. burnetii* also forms spores that may survive for months in the environment in an area where animals have been present, representing a source of infection for persons without any evident contact with animals (*2*). A self-limited febrile illness, Q fever has the major signs and symptoms of atypical pneumonia and hepatitis. Diagnosis is based on serologic test results. Recently, outbreaks of Q fever have been described in urban areas (*3*,*4*), affecting people without any evident risk factor. We describe an outbreak of Q fever in Como, northern Italy, which affected 133 persons.

From January 2003 to February 2003, over a 5-week period, 16 men and 1 woman from the prison in Como were admitted to the local hospital with acute pneumonia. At the same time, a 26-year-old man and a 79-year-old woman living in the same area were admitted with the same diagnosis. On admission, all the patients had a high-grade fever and reported a dry cough; 80.0% of patients had a headache, and 70.0% of patients complained of fatigue and weakness. In a few patients, nausea and abdominal pain developed after they were hospitalized. Physical examination of the lungs showed minimal auscultatory abnormalities. Hemoptysis was observed in four patients. Routine blood examinations were performed. In all patients, the leukocyte count was normal, with an increase in the erythrocyte sedimentation rate and elevated C-reactive protein levels. The transaminase levels were elevated in eight (47.0%) of patients, usually two to three times normal values. Radiographic findings were nonspecific and differed greatly among the patients, who exhibited single and multiple opacities, diffuse interstitial pneumonia, and pleural effusion (in one patient). Bilateral involvement was seen in five (29.4%) patients. All patients received empirical antimicrobial therapy with a β-lactam antimicrobial drug in association with a macrolide or an advanced fluoroquinolone, following the guidelines for community-acquired pneumonia. Patients improved clinically in 48 to 72 hours after antimicrobial drug therapy was started: the fevers resolved and the inflammatory indexes and liver enzyme levels returned to normal.

An epidemiologic investigation was started by the Department of Prevention, Azienda Sanitaria Locale, of Como, after it was notified that six patients with acute pneumonia of unknown cause had been admitted to St. Anna Hospital during a 6-day period (January 11–17, 2003); all six patients were men from the Como prison. Investigation showed that the case-patients resided in different sections of the prison in different rooms; thus, human-to-human transmission was excluded. After exclusion of *Streptococcus pneumoniae,* *Legionella* spp., *Mycoplasma pneumoniae,* and *Chlamydia pneumoniae* as etiologic agents, the diagnosis of Q fever was made on the basis of positive serologic results for *C. burnetii* from immunofluorescence assay. Further investigation was directed to determine the source of the epidemic, with a focus on animals in the area surrounding the prison. We identified two flocks of sheep and other animals that grazed in the meadows next to the prison from December 2002 to January 2003, in an annual migration pattern, and a colony of pigeons nesting in the prison. The first flock of animals was composed of 950 sheep; 50 goats and 4 dogs accompanied them. The Laboratory of the Zooprofilattico Institute, Brescia, tested 80 animals from this flock, including both males and females, chosen randomly. All four dogs were tested. All the animals tested (sheep, goats, and dogs) were negative for *C. burnetii*. The second flock included 748 animals (sheep and goats) and 4 dogs. All these animals were tested because the biological specimens, collected for another purpose, were available. In this flock, 255 of 748 animals were positive for *C. burneti,i* and 2 of the 4 dogs were positive, for a prevalence rate of 34.2%. We know that a parturient sheep died on December 31, 2002. The source of the epidemic was declared extinct on March 5, 2003, on the basis of a negative result of DNA amplification by polymerase chain reaction assay conducted on animals’ milk, urine, and feces after antimicrobial drug treatment of the infected animals.

At the same time, in collaboration with physicians of general medicine in the area, we actively sought patients with pneumonia of unknown cause living in the urban area close to the meadows where the flock grazed. Since the beginning of the epidemic, 133 cases of acute Q fever, defined as clinical symptoms (high grade fever, dry cough, auscultatory abnormalities, arthromyalgia, fatigue) plus positive serologic results (immunoglobulin [Ig] G phase II >1:64; values were included between 1:64 and 1:1024), were reported to the Department of Prevention, ASL, in Como. Of these, 59 were prisoners, 37 were prison officers, 33 were persons living in the area in which the flock traveled**,** and 4 were personnel of the Veterinarian Service, who participated in performing the autopsy on the sheep. Analysis of the data showed a prevalence of disease in the prisoners of 10.8% (59/547), comparable to the prevalence obtained in the guards, 16.5% (37/224) and significantly lower than that for the residents, 3.2% (33/1,025). These differences could be ascribed to the prison’s being situated in a natural setting, with the flock grazing for 1 month in the meadows nearby.

*C. burnetii* is an infrequent cause of community-acquired pneumonia in our region. These data suggest that the infected sheep were the source of this large outbreak. None of the patients had any contact with animals, except for the Veterinarian Service personnel, which suggests airborne transmission of infected dust particles from contaminated soil, favored by the dry weather recorded in that period. To our knowledge, this is the second outbreak of Q fever reported in northern Italy (*5*). Since 1999, neither the Department of Prevention nor the Veterinary Service had received any reports of Q fever in Como. In Italy, the total number of cases of rickettsial diseases, which includes Q fever, reported to the Ministry of Health was 769 in 2002 and 739 in 2001, with 5 and 13 cases, respectively, from Lombardia, which includes Como. This Q fever epidemic in Como is thus an exceptional event in our area.

The collaboration between epidemiologists and veterinarians of the Department of Prevention and staff from Saint Anna Hospital allowed us to share epidemiologic and medical information, which proved useful in diagnosing the outbreak and treating patients. Our experience emphasizes the necessity of a greater awareness of this occupational zoonosis in areas with a high rate of urbanization.
